# LncRNA-RMRP Acts as an Oncogene in Lung Cancer

**DOI:** 10.1371/journal.pone.0164845

**Published:** 2016-12-01

**Authors:** Qingjun Meng, Mingming Ren, Yanguang Li, Xiang Song

**Affiliations:** Department of thoracic surgery, CangZhou central hospital, CangZhou, Hebei, China; University of South Alabama Mitchell Cancer Institute, UNITED STATES

## Abstract

Accumulating studies have demonstrated that long noncoding RNAs (lncRNAs) act a crucial role in the development of tumors. However, the role of lncRNAs in lung cancer remains largely unknown. In this study, we demonstrated that theexpression of RMRP was upregulated in lung adenocarcinoma tissues compared to the matched adjacent normal tissues. Moreover, of 35 lung adenocarcinoma samples, RMRP expression was upregulated in 25 cases (25/35; 71.4%) compared to the adjacent normal tissues. We also showed that RMRP expression was upregulated in lung adenocarcinoma cell lines (A549, SPC-A1, H1299 and H23) compared to the bronchial epithelial cell line (16HBE). Ectopic expression of RMRP promoted lung adenocarcinoma cell proliferation, colony formation and invasion. In addition, overexpression of RMRP inhibited the miR-206 expression in the H1299 cell and increased the KRAS, FMNL2 and SOX9 expression, which were the target genes of miR-206. Re-expression of miR-206 reversed the RMRP-induced the H1299 cell proliferation and migration. Our data proved that RMRP acted as an oncogene LncRNA to promote the expression of KRAS, FMNL2 and SOX9 by inhibiting miR-206 expression in lung cancer. These data suggested that RMRP might serve as a therapeutic target in lung adenocarcinoma.

## Introduction

Lung cancer is the leading cause of tumor death worldwide, with about 1,400,000 deaths every year [1–4]. Non-small cell lung cancer (NSCLC), including squamous cell carcinoma and adenocarcinoma, accounts for about 85% of lung cancer [5–8]. The five-year overall survival rate of late-stage NSCLC patients is only 5–20% [9–13]. Despite the improvements in surgery, chemotherapy and radiotherapy, the overall survival of NSCLC is still not encouraging [14–16]. Therefore, it is important to identify noninvasive, new prognostic biomarkers for the NSCLC to develop new therapeutic target for NSCLC.

Long noncoding RNAs (lncRNAs) are noncoding RNAs longer thannucleotides. [17–20]. Increasing evidences show that lncRNAs play a critical role in the cell biology such as cell proliferation, metabolism, differentiation, development, invasion, migration and apoptosis [21–25]. Recently, many lncRNAs are proved to be deregulated in several tumors such as gastric cancer, ovarian carcinoma, colorectal tumor, hepatocellular carcinoma and also lung cancer [19, 24, 26–28]. However, the expression, function and roles of lncRNAs are still not well studied.

RMRP is a long non-coding RNA that was expressed in vairous murine and human tissues [29]. Previous study showed that the expression of RMRP was deregulated in gastric cancer [30]. Moreover, Shao et al [31]. showed that RMRP expression level was downregulated in gastric cancer tissues and gastric dysplasia. RMRP suppressed the expression of miR-206 and regulated the cell cycle by modulating Cyclin D2 expression in gastric cancer cell. However, the role of RMRP in lung cancer is still known. In this study, we demonstrated that RMRPexpression was upregulated in lung adenocarcinoma tissues and cell lines. Ectopic expression of RMRP promoted lung adenocarcinoma cell proliferation, colony formation and invasion. Overexpression of RMRP inhibited the miR-206 expression in the H1299 cell and increased the expression of KRAS, FMNL2 and SOX9, which were the target genes of miR-206. Re-expression of miR-206 reversed the RMRP-induced the H1299 cell proliferation and migration.

## Materials and Methods

### Tissue samples and cell lines cultured and tranfected

All lung adenocarcinoma tissues and matched adjacent normal tissues were handled at the CangZhou central hospital. All lung adenocarcinoma patients did not receive radiotherapy or chemotherapy before surgery. All patients gave their written informed consent to participate in this study and the protocal of this study were approved by the Ethics Committee of CangZhou central hospital. The lung adenocarcinoma cell lines (A549, SPC-A1, H1299 and H23) and 16HBE (bronchial epithelial cell line) were bought from the cell bank of the Chinese Academy of Sciences (Shanghai, China). These cell lines were kept in the RPMI 1640 medium. PDNA3.1-RMRP vector and control vector were synthesized by the GenePharma (Shanghai, China). Cell tranfection was performed by the Lipofectamine 2000 reagent according to the manufacturer’s instructions.

### Quantitative real-time PCR

Total RNAs were extracted from cells or tissues by the Trizol reagent (Invitrogen, USA) following to the manufacturer’s instruction. Real-time PCR was done to measure the lncRNA and mRNA expression using the SYBR Green PCR kit on the ABI 7300 system (Applied Biosystems, USA) following to the manufacturer’s information. GAPDH and 18S rRNA was used as the control for mRNA or lncRNA expression respectively. The primer sequences were used as following: LncRNA-RMRP, 5’-ACTCCAAAGTCCGCCAAGA-3’ (forward) and 5’-TGCGTAACTAGAGGGAGCTGAC-3’ (reverse); GAPDH, 5’-GGGAGCCAAAAGGGTCAT 3’ (forward) and reverse primer: 5’-GAGTCCTTCCACG ATACCAA 3’ (reverse). 18S rRNA, 5’-ACACGGACAGGATTGACAGA-3’ (forward) and 5’-GGACATCTAAGGGCATCACA-3’ (reverse).

### Cell proliferation, colony formation and invasion assays

The cells were cultured in the 96-well plate with 1x10^4^ per well. MTT (5 mg/ml; Sigma, USA) were put into the each well for addition 4 hours. Optical density (OD) was measured by deteting the absorbance at the 490 nm using the microplate reader (Bio-Rad, USA). Cells were treated with PDNA3.1-RMRP or control vector for 24 hours and then cultured for colony formation in the 6-well plate for 14 days. The colonies were fixed with paraformaldehyde and then stained with crystal violet. Cells invasion were evaluated using Matrigel chambers (BD Biosciences, United Kingdom) following with the manufacturer’s information. Cell was cultured in the upper chamber in DMEM medium withour serum, and the lower chamber supplemented with FBS. After 24 hours, the noninvading cells were discarded using the cotton swab and the bottom cells were fixed with paraformaldehyde and stained with crystal violet.

### Western blot assay

Protein was extracts from cell or tissue using the cell lysis reagent and measured by the BCA kit (Pierce, USA) according to the instruction’s information. Protein was separated from 12% SDS—PAGE and then transferred to the PVDF membranes. The blot was incubated with primary antibody (KRAS, FMNL2 and SOX9, Abcam) for 12 hours at 4°C and then measured with secondary antibody with HRP-conjugated (1:10000). Signal was measured by using ECL (Millipore). GAPDH was used as the control for normalization.

### Statistical analysis

Data was shown as the mean±SD and statistical analyse was performed using the SPSS 17.0 software (IBM SPSS, USA). The difference between two groups was assessed by the Student t-test and the difference between more than two groups was estimated by the one-way ANOVA. P< 0.05 was considered statistically significant.

## Results

### The expression of RMRP was upregulated in the lung adenocarcinoma tissues

**Fig 1:**
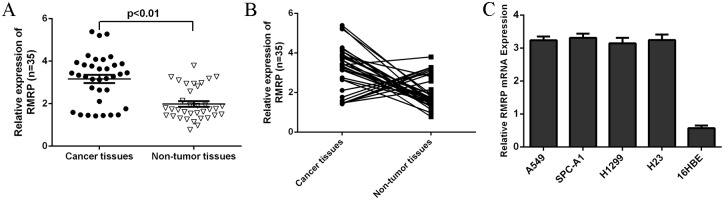
The expression of RMRP was upregulated in the lung adenocarcinoma tissues. (A) RMRP expression was measured in the lung adenocarcinoma tissues and the matched adjacent normal tissues using qRT-PCR. (B) The RMRP was upregulated in 25 cases (25/35; 71.4%) compared to the adjacent normal tissues. (C) The RMRP expression was upregulated in lung adenocarcinoma cell lines (A549, SPC-A1, H1299 and H23) compared to the bronchial epithelial cell line (16HBE).

As shown in Fig 1A, RMRP expression was upregulated in the lung adenocarcinoma tissues compared to the matched adjacent normal tissues. Moreover, of 35 lung adenocarcinoma samples, RMRP was upregulated in 25 cases (25/35; 71.4%) compared to the adjacent normal tissues (Fig 1B). In addition, the RMRP expression was upregulated in lung adenocarcinoma cell lines (A549, SPC-A1, H1299 and H23) compared to the bronchial epithelial cell line (16HBE) (Fig 1C).

### Ectopic expression of RMRP enhanced lung adenocarcinoma cell proliferation, colony formation and invasion

**Fig 2:**
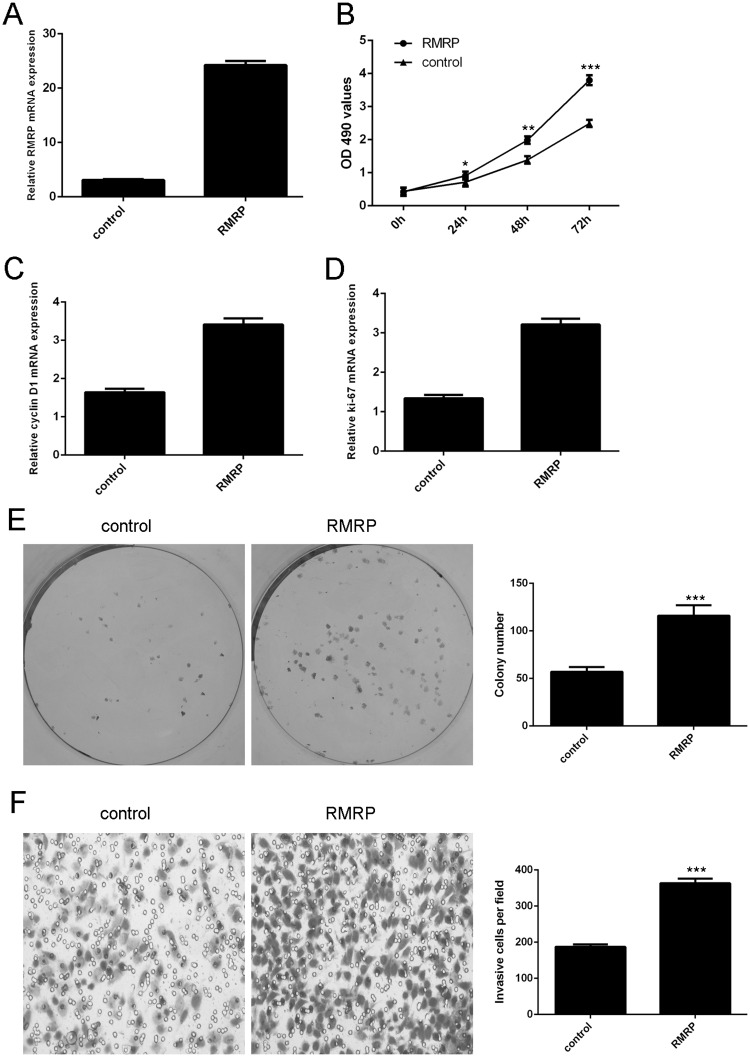
Ectopic expression of RMRP promoted lung adenocarcinoma cell proliferation, colony formation and invasion. (A) The expression of RMRP was measured in the H1299 cell after treated with RMRP vector. (B) Ectopic expression of RMRP promoted H1299 cell proliferation. (C) Overexpression of RMRP enhanced the cyclin D1 expression in the H1299 cell. (D) Ectopic expression of RMRP promoted ki-67 expression in the H1299 cell. (E) Overexpression of RMRP promoted the H1299 cell colony formation. (F) Overexpression of RMRP enhanced the H1299 cellinvasion. *p<0.05, **p<0.01 and ***p<0.001.

The expression of RMRP was significantly enhanced after treated with RMRP vector (Fig 2A). Ectopic expression of RMRP promoted lung adenocarcinoma cell line H1299 cell proliferation (Fig 2B). In line with this, overexpression of RMRP enhanced the expression of cyclin D1 and ki-67 (Fig 2C and 2D). Overexpression of RMRP promoted the H1299 cell colony formation (Fig 2E). Ectopic expression of RMRP increased the H1299 cell invasion (Fig 2F).

### miR-206 expression was downregulated in the lung adenocarcinoma tissues

**Fig 3:**
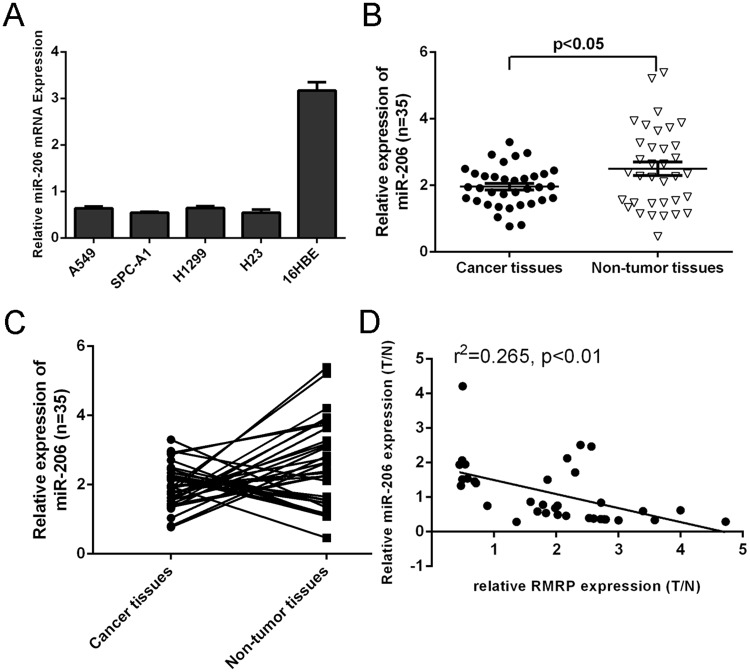
miR-206 expression was downregulated in the lung adenocarcinoma tissues. (A) The miR-206 expression was measured in lung adenocarcinoma cell lines (A549, SPC-A1, H1299 and H23) and the bronchial epithelial cell line using qRT-PCR. (B) The miR-206 expression was detected in lung adenocarcinoma tissues and the matched adjacent normal tissues by using qRT-PCR. (C) miR-206 expression was downregulated in 21 cases (21/35; 60%) compared to the adjacent normal tissues. (D) The expression of RMRP was negative correlated with the expression of miR-206 in lung adenocarcinoma tissues.

miR-206 expression was downregulated in lung adenocarcinoma cell lines (A549, SPC-A1, H1299 and H23) compared to the bronchial epithelial cell line (Fig 3A). As shown in Fig 3B, miR-206expression was downregulated in lung adenocarcinoma tissues compared to the matched adjacent normal tissues. Moreover, of 35 lung adenocarcinoma samples, miR-206 expression was downregulated in 21 cases (21/35; 60%) compared to the adjacent normal tissues (Fig 3C). As shown in the Fig 3D, the expression of RMRP was negative correlated with the expression of miR-206 in lung adenocarcinoma tissues.

### RMRP suppressed expression of miR-206 and increased the expression of KRAS, FMNL2 and SOX9

**Fig 4:**
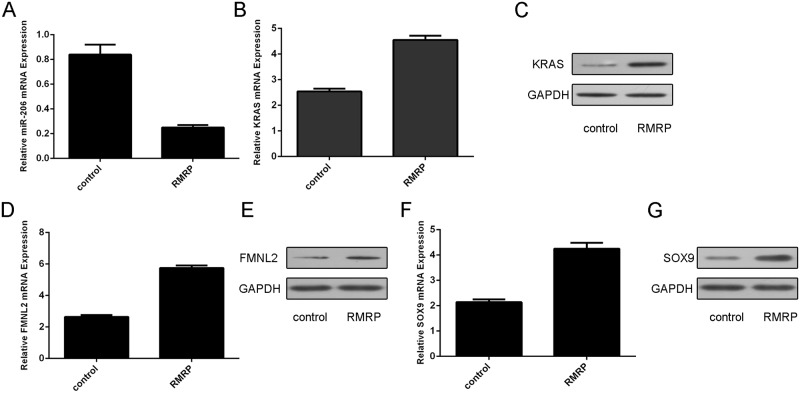
RMRP suppressed expression of miR-206 and increased the expression of KRAS, FMNL2 and SOX9. (A) Overexpression of RMRP inhibited theexpression of miR-206 in the H1299 cell. (B) Ectopic expression ofRMRP promoted the KRAS mRNA expression in the H1299 cell. (C) The protein expression of KRAS was determined using western blot. (D) Ectopic expression ofRMRP promoted the FMNL2 mRNA expression in the H1299 cell. (E) The protein expression ofFMNL2 was determined using western blot. (F) Ectopic expression ofRMRP promoted the SOX9 mRNA expression in the H1299 cell. (G) The protein expression ofSOX9 was determined using western blot.

miR-206 was proved to act as a tumor suppressor miRNA in lung cancer through targeting the KRAS, FMNL2 and SOX9. Overexpression of RMRP inhibited theexpression of miR-206 in the H1299 cell (Fig 4A). Moreover, ectopic expression of RMRP promoted the expression of KRAS, FMNL2 and SOX9 (Fig 4B–4G) in H1299 cell.

### RMRP exhibited an oncogenic activity through targeting miR-206

**Fig 5:**
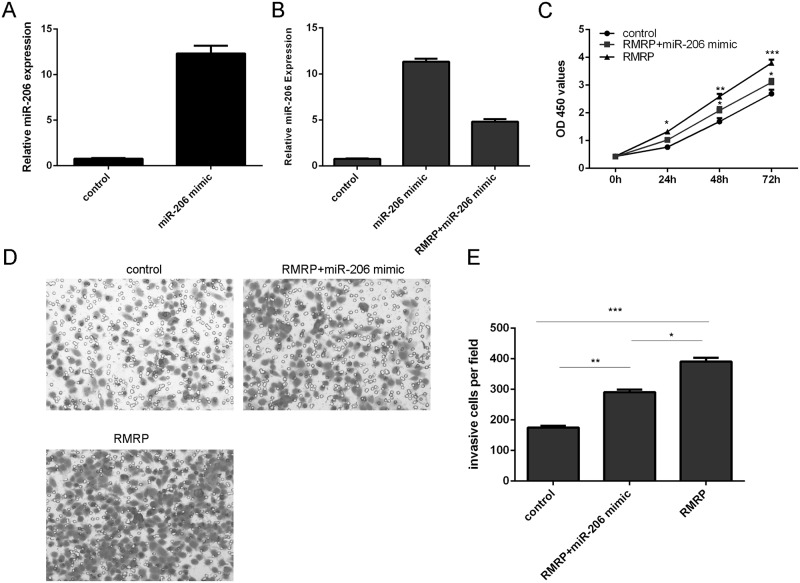
RMRP exhibited an oncogenic activity through targeting miR-206. (A) miR-206 expression was upregulated in H1299 cell after treated with the miR-206 mimic. (B) miR-206 expression was decreased in the RMRP-induced H1299 cell after treated with RMRP vector. (C) Restoration of miR-206 suppressed cell proliferation in the RMRP-induced H1299 cell after treated with miR-206 mimic. (D) Restoration of miR-206 inhibited the cell invasion in the RMRP-induced H1299 cell after treated with miR-206 mimic.*p<0.05, **p<0.01 and ***p<0.001.

miR-206expression was significantly upregulated in H1299 cell after treated with the miR-206 mimic (Fig 5A). miR-206 expression was decreased in H1299 cell after treated with RMRP vector (Fig 5B). CCK8 proliferation assay demonstrated that restoration of miR-206 suppressed cell proliferation in H1299 cell after treated with miR-206 mimic (Fig 5C). Moreover, invasion assay showed that restoration of miR-206 inhibited the cell invasion in H1299 cell after treated with miR-206 mimic (Fig 5D).

## Discussion

In this study, we demonstrated that RMRP expression was upregulated in lung adenocarcinoma tissues compared to the matched adjacent normal tissues. Moreover, of 35 lung adenocarcinoma samples, RMRP expression was upregulated in 25 cases (25/35; 71.4%) compared to the adjacent normal tissues. We also showed that RMRP expression was upregulated in lung adenocarcinoma cell lines (A549, SPC-A1, H1299 and H23) compared to the bronchial epithelial cell line (16HBE). Ectopic expression of RMRP increased lung adenocarcinoma cell proliferation, colony formation and invasion. In addition, overexpression of RMRP inhibited miR-206 expression in the H1299 cell and increased the expression of KRAS, FMNL2 and SOX9, which were the target genes of miR-206. Re-expression of miR-206 reversed the RMRP-induced the H1299 cell proliferation and migration. These data suggested that RMRP acted as an oncogene LncRNA to promote the KRAS, FMNL2 and SOX9 by inhibiting miR-206 expression in the lung cancer.

LncRNAs play critical roles in the development of various cancers [32–34]. Previous study showed that RMRP was deregulated in gastric cancer [30]. Moreover, Shao et al [31]. demonstrated that RMRP expression was downregulated in gastric cancer tissues and gastric dysplasia. RMRP inhibited the expression of miR-206 and regulated the cell cycle through modulating Cyclin D2 expression in gastric cancer cell. However, the role of RMRP in lung cancer is still uncovered. In this study, we firstly measured RMRP expression in 35 pairs of lung adenocarcinoma tissues and matched adjacent normal tissues. Our results showed that RMRP expression was upregulated in lung adenocarcinoma tissues compared to the matched adjacent normal tissues. Of 35 lung adenocarcinoma samples, RMRP expression was upregulated in 25 cases (25/35; 71.4%) compared to the adjacent normal tissues. In line with this, RMRP expression was upregulated in lung adenocarcinoma cell lines compared to the bronchial epithelial cell line. Moreover, ectopic expression of RMRP promoted lung adenocarcinoma cell proliferation, colony formation and invasion. These data suggested that RMRP acted as aoncogeniclncRNA in lung cancer.

LncRNAs regulate gene expression epigenetically through competing for shared the miRNA response elements, therefore decreasing the binding of miRNA to its target genes [35–38]. Previous study showed that RMRP increased aggressive gastric cancer by regulating Cyclin D2 as the ceRNA for miR-206 [31]. In line with this, we demonstrated that overexpression of RMRP suppressed miR-206 expression in lung adenocarcinoma cell. In addition, our data showed that ectopic expression of RMRP promoted theexpression of KRAS, FMNL2 and SOX9 in lung adenocarcinoma cell. Previous study suggested that miR-206 expression was downregulated in gastric cancer tissues compared to the normal adjacent mucosa [39–42]. Zhang et al [43]. demonstrated that miR-206 expression was downregulated in non small cell lung cancer tissues compared with adjacent normal tissues. Overexpression of miR-206 suppressed the cell proliferation, invasion and migration of NSCLC cells through targeting SOX9. In addition, Ren et al [44]. showed that miR-206 expression was decreased in colorectal cancer (CRC) tissues and associated with lymphatic metastasis, differentiation and serosal invasion. Overexpression of miR-206 inhibited CRC cell proliferation and increased the cell apoptosis by inhibiting FMNL2 expression. Keklikoglou et al. demonstrated that miR-206 was abrogated in pancreatic ductal adenocarcinoma cell lines and tissues. They showed that miR-206 suppressed the pancreatic ductal adenocarcinoma cell cycle progression, migration, invasion and proliferation by targeting KRAS and annexin a2 (ANXA2) [45]. In our study, we also demonstrated that ectopic expression of RMRP promoted the expression of KRAS, FMNL2 and SOX9 in lung adenocarcinoma cell, which were the direct taget gene of miR-206. Furthermore, RMRP exhibited an oncogenic activity through targeting miR-206 in lung adenocarcinoma cell.

In conclusion, we demonstrated that RMPR expression was upregulated in lung adenocarcinoma tissues and overexpression of RMRP promoted lung adenocarcinoma cell proliferation, colony formation and invasion. RMRP exhibited an oncogenic activity through targeting miR-206 in lung adenocarcinoma cell. Therefore, RMRP might serve as a therapeutic target in lung adenocarcinoma.
